# Renal protective effect of selenium on cisplatin-induced nephrotoxicity

**DOI:** 10.12861/jrip.2012.11

**Published:** 2012-01-01

**Authors:** Ali Ghorbani

**Affiliations:** ^1^Department of Nephrology, Golestan Hospital, Ahvaz Jundishapur University of Medical Sciences, Ahvaz, Iran

**Keywords:** Selenium, Cisplatin, Nephrotoxicity, Oxidative stress

**Implication for health policy/practice/research/medical:**

Selenium supplementation reduces the products of oxidative stress in kidney failure patients and can be protective against cisplatin nephrotoxicity.

Recently much attention has been directed toward kidney protective efficiency of selenium. In the article published by Hemati *et al* entitled, the effects of vitamin E and selenium on cisplatin-induced renal toxicity in cancerous patients treated with cisplatin-based chemotherapy, they found that, selenium can be used to reduce cisplatin-induced nephrotoxicity (1). Kidney injury is common following cisplatin treatment. Recently in a double-blind controlled randomized clinical trial, we studied 122 cancerous patients who were candidate to receive chemotherapy regimens consisting cisplatin. In this study, we found that, selenium could prevent cisplatin-induced acute kidney injury, when it is added to hydration therapy in cancerous patients (2). Furthermore, the results of the study conducted by Randjelovic *et al*, showed that selenium attenuates oxidative-stress-associated kidney injury by reducing oxygen free radicals and lipid peroxidation in gentamicin-treated rats (3). Indeed, gentamicin-induced tissue injury was mediated through oxidative reactions (1-3). Selenium is a trace element that participates as a cofactor in several enzymes, one of them is participation in the regulation of enzymatic antioxidant defenses (4). It was established that, selenium supplementation in kidney failure patients, reduces the products of oxidative stress (2-4). Moreover, in the study of adriamycin -induced kidney damage in rats, Taskin *et al* showed that selenium is protective *in vivo* against Adriamycin-induced renal toxicity through the restoration of total antioxidant-oxidant status, which prevented mitochondrial damage (5). Recent studies revealed that plasma selenium level have been decreased in patients with acute renal injury (4,6). As well, low serum selenium level is a frequent finding in patients with chronic kidney disease (6-8). Though, to date, few investigations have studied the association of low serum selenium level and morbidity and mortality in kidney failure patients, the available data lend further evidence for the attribution of selenium in its kidney protective effect (5-8). In this regard, to better understanding the selenium renal protective properties, more experimental rat models or clinical studies are suggested.

## Author’s contribution

AG is the single author of the manuscript.

## Conflict of interests

The author declared no competing interests.

## Ethical considerations

Ethical issues (including plagiarism, data fabrication, double publication) have been completely observed by the author.

## Funding/Support

None.
